# The Scandinavian pre-hospital physician and documentation of clinical data in the trauma patient

**DOI:** 10.1186/1757-7241-21-S1-S15

**Published:** 2013-05-28

**Authors:** AJ Kruger

**Affiliations:** 1 Norwegian Air Ambulance Foundation & St. Olavs Hospital, Trondheim, Norway

## Background

Documentation of clinical data in the pre-hospital emergency care is important for research purposes. Collecting complete patient level data can be challenging as time is limited and patient management must have priority. In the current study we aimed to examine the ability of Scandinavian pre-hospital physicians to record of fourteen clinical variables during pre-hospital trauma missions.

## Method

Prospective study of all patients attended by sixteen physician-staffed pre-hospital services in Scandinavia for a 4 week period (2 weeks summertime, 2 weeks wintertime). Total population covered by participating services was approximately 12,5 million.

All patient encounters were recorded by the physician on-call and plotted into a predefined web-based database. We requested medical complaint as well as patient level data including age, gender and seven physiological variables (Glasgow Coma Scale, heart rate, respiratory rate, heart rhythm, pain, systolic blood pressure and oxygen saturation) at initial patient contact and at patient handover. Patients were classified into two categories; not severely injured, or severely injured (recept of advanced procedure and/or recept of advanced medication and/or severely physiologically de-arranged). Patient care time was defined as on-scene time+ transportation time.

Results are presented using descriptive statistics.

## Results

2256 missions with patient encounter were recorded during the study period. 519 of these were trauma missions (Norway: 112, Sweden:46, Denmark: 311 and Finland: 50). 131 patients were severely ill or injured. Median patient care time was 26 (Norway), 20 (Sweden),17(Denmark) and 12 (Finland) minutes for non-severely injured patients. For missions involving a severely injured patient durations were longer; 38 (Norway), 44 (Sweden), 29,5(Denmark) and 53 minutes (Finland).

The completeness rate of documentation of clinical variables is depicted in figure 1 and 2. The rate of completeness is generally higher in missions involving severely injured patients. Generally, the Danish services have a lower completeness rate than the other Scandinavian services.

## Conclusion

A near 100% documentation of the requested clinical variables in this study can be achieved, also in missions involving a severely injured patient. The reason for in-complete documentation should be addressed and efforts should be taken to increase overall completeness rate.

